# The Role of Mitochondria in Polycystic Kidney Disease

**DOI:** 10.3390/ijms27114774

**Published:** 2026-05-26

**Authors:** Yuhe Wang, Jianhua Mao, Fei Liu

**Affiliations:** Department of Nephrology, Children’s Hospital, Zhejiang University School of Medicine, National Clinical Research Center for Child and Adolescents’ Health and Diseases, Hangzhou 310052, China; 22518722@zju.edu.cn (Y.W.); maojh88@zju.edu.cn (J.M.)

**Keywords:** polycystic kidney disease, mitochondria, metabolic reprogramming, oxidative stress, mitophagy, cell signaling

## Abstract

Polycystic kidney disease (PKD) is a genetic disorder characterized by renal cyst formation and progressive renal dysfunction, where inflammation, immune responses, and metabolic dysregulation critically drive disease progression, while emerging evidence increasingly links its pathogenesis to mitochondrial dysfunction. Mitochondria, central to cellular energy production, metabolism, and redox homeostasis, exhibit profound abnormalities in PKD, contributing to disease pathogenesis. Current evidence on mitochondrial mechanisms driving PKD progression includes metabolic reprogramming, oxidative stress, disrupted mitochondrial dynamics, and impaired mitophagy. Polycystic kidney disease is caused by mutations in the *PKD1* or *PKD2* genes, which encode polycystin 1 and polycystin 2. The formation of dysfunctional polycystins (PC1/PC2) is a key event in the pathogenesis of this disease, triggering impaired calcium signaling, increased production of mitochondrial reactive oxygen species (ROS), and reduced oxidative phosphorylation, thereby promoting cyst growth and fibrosis. Key signaling pathways such as mTORC1 hyperactivation, AMPK suppression, and disrupted calcium homeostasis further exacerbate mitochondrial defects. Emerging therapeutic strategies targeting mitochondrial pathways, such as mitochondrial antioxidants, modulators of mitophagy, calcium signaling regulators, and metabolic reprogramming agents, show promise in preclinical models. However, challenges remain in translating these findings to clinical applications, including drug specificity and minimizing off-target effects. This review underscores mitochondria as pivotal players in PKD pathogenesis and highlights their potential as therapeutic targets to mitigate cystogenesis and disease progression.

## 1. Introduction

Polycystic kidney disease (PKD) is a genetic disorder characterized by the formation and progressive enlargement of fluid-filled cysts in the kidneys. These cysts compress normal renal tissue, leading to kidney enlargement, functional decline, and ultimately end-stage renal disease (ESRD) [1,2]. According to inheritance pattern, PKD is broadly classified into two types, Autosomal Dominant Polycystic Kidney Disease (ADPKD) and Autosomal Recessive Polycystic Kidney Disease (ARPKD). ADPKD is mainly caused by mutations in *PKD1* and *PKD2*, which encode polycystin-1 (PC1) and polycystin-2 (PC2). Mutations in *PKD1* account for 78% of ADPKD cases while mutations in *PKD2* account for 15% of the ADPKD cases [3]. *PKD1* mutations are associated with earlier onset and more severe disease. These proteins localize to primary cilia and regulate calcium signaling and cellular homeostasis [4,5]. ARPKD is rarer and much more severe than ADPKD, occurring in 1 in 20,000 births. ARPKD is primarily caused by mutations in *PKHD1*, which encode a large ciliary membrane protein called Fibrocystin or Polyductin (FPC) [6]. ADPKD and ARPKD, while genetically distinct, share overlapping pathways involving mitochondrial dysfunction, metabolic reprogramming, and ciliary defects.

Mitochondria are indispensable organelles critical for maintaining cellular homeostasis and enabling life-sustaining processes. They are not only responsible for energy production, but also play important roles in metabolic regulation, stress response, and biosynthesis [7]. Mitochondria also regulate cell survival, controlling apoptosis by releasing cytochrome c into the cytoplasm and activating caspases that execute cell death [8]. Additionally, mitochondria buffer cytosolic Ca^2+^ to modulate signaling and harbor their own mitochondrial DNA (mtDNA) encoding electron transport chain (ETC) components, underscoring their central role in cell survival and health [9]. The kidney is an organ rich in mitochondria and high energy metabolism. Mitochondrial dysfunction and stress responses are deeply involved in multiple kidney disease progression, such as chronic kidney disease (CKD), diabetic kidney disease (DKD) and acute kidney injury (AKI) [10]. Mitochondrial dysfunction is increasingly recognized as a central contributor of pathogenesis in kidney diseases, particularly in PKD, basically including four aspects, metabolic reprogramming, oxidative stress, mitochondrial dynamics and mitophagy defects. In addition, multiple studies highlight that PC1 and PC2 may regulate mitochondria function directly [11]. Importantly, progressive cyst enlargement compresses the surrounding renal parenchyma, impairing local perfusion and oxygen delivery. Consequently, cyst-induced tissue hypoxia further compromises mitochondrial respiratory chain function, exacerbating reactive oxygen species generation and fueling a vicious cycle of mitochondrial damage, inflammation, and fibrosis [12]. This interplay between structural compression and metabolic vulnerability underscores the central role of mitochondria in PKD progression.

While the primary genetic lesions in PKD are well defined, the molecular mechanisms connecting ciliary proteins, mitochondrial dysfunction, and cystogenesis remain incompletely understood. Increasing evidence supports mitochondria as critical mediators linking genetic mutations to cellular dysfunction and disease progression. Therefore, a comprehensive understanding of mitochondrial perturbations in PKD is essential for identifying actionable therapeutic targets. This review systematically synthesizes current evidence on mitochondrial abnormalities underlying PKD pathogenesis and discusses emerging mitochondria-targeted therapeutic strategies, along with future translational perspectives.

A comprehensive literature search was performed in PubMed, Web of Science, and Scopus databases using keywords including “polycystic kidney disease,” “mitochondria,” “metabolic reprogramming,” “oxidative stress,” “mitophagy,” and “therapeutic targets.” The search covered publications from database inception to January 2026. A total of 133 original articles and reviews were included for analysis.

## 2. Mitochondrial Mechanisms in Polycystic Kidney Disease

### 2.1. Role of Energy Metabolism in Mitochondria and Its Impact on Renal Cell Function

Mitochondria are central to energy production and cellular homeostasis in renal cells. In ADPKD, a broad reprogramming of multiple metabolic pathways is altered, culminating in the mitochondrial energy metabolism [13]. Core mitochondrial energy pathways include oxidative phosphorylation (OXPHOS), fatty acid oxidation (FAO) and tricarboxylic acid cycle (TCA).

#### 2.1.1. Drivers of the Warburg Effect in PKD

Increased glycolysis, defective OXPHOS and FAO have been observed both in vitro and in vivo in animal models of ADPKD and in tissues from patients with ADPKD [1,2]. In PKD, cyst-lining cells shift from OXPHOS to aerobic glycolysis (Warburg effect), increased cellular glucose uptake and lactate production, instead of pyruvate oxidation. A current systems biology analysis also found that gene expression profiles of *PKD1* renal cysts were consistent with the Warburg effect [14]. In addition, mammalian target of rapamycin complex 1 (mTORC1) may activate two key transcription factors: MYC and hypoxia-inducible factor 1 subunit alpha (HIF-1α), leading to Warburg Effect by causing increased expression of genes in aerobic glycolysis and inhibiting the mitochondrial TCA cycle and OXPHOS [14]. mTORC1 hyperactivation may stabilize HIF-1α, which drives the expression of genes promoting aerobic glycolysis, upregulates glucose transporter 1 (GLUT1) [15] and lactate dehydrogenase (LDH), diverting pyruvate to lactate instead of mitochondrial oxidation [16]. In renal cells, the switch to glycolysis compromises energy-intensive processes like sodium–potassium ATPase activity, impairing tubular fluid regulation and promoting cyst expansion [17,18].

#### 2.1.2. Suppression of Oxidative Phosphorylation

Recent *PKD1*-edited cell models show that homozygous knockout and compound heterozygous mutations exhibit reduced mitochondrial mass, significantly decreased basal and maximal respiration, and impaired oxidative phosphorylation, confirming that loss of polycystin-1 causes defective mitochondrial biogenesis and OXPHOS [19]. Consistent with these findings, OXPHOS is suppressed in PKD and this metabolic impairment may be associated with decreased expression of peroxisome proliferator-activated receptor γ coactivator 1α (PGC1α). PGC1α is regulated by AMP-activated protein kinase (AMPK) by multiple mechanisms and affects transcription factor peroxisome proliferator-activated receptor-α (PPARα) [14]. AMPK is the primary sensor of cellular energy stores and it is activated in the presence of high AMP levels [20]. In PKD, AMPK activity is suppressed due to the high ATP level generated via glycolysis and the regulation of the LKB1/AMPK axis by the extracellular signal-regulated kinases (ERKs) [1,2]. The reduction in AMPK activity fails to inhibit mTORC1 via dual phosphorylation of tuberous sclerosis complex 2 (TSC2) and the regulatory-associated protein of mTOR. Therefore, mTORC1 is also involved in regulation of PGC1α and PPARα and both PGC1α and PPARα promote energy metabolism by affecting fatty FAO and OXPHOS [14,15]. Also, mitochondria in PKD cells exhibit fragmented morphology and disorganized cristae, reducing OXPHOS efficiency [21]. Defective OXPHOS leads to the generate excess mitochondrial ROS (mtROS), which oxidizes mtDNA, lipids, and proteins, impairing mitochondrial enzymes, greatly impacting renal function [22,23].

#### 2.1.3. Impaired Fatty Acid Oxidation and Lipotoxicity

Downregulation of key enzymes in TCA cycle reduces NADH production, crippling ETC activity [14]. Reduced expression of Complex I (NADH dehydrogenase) and Complex III disrupts the proton gradient, lowering ATP synthesis. FAO defect has also been proved as a key PKD feature. Impaired FAO is caused by the upregulation of microRNA-17 (miRNA), which downregulates the expression levels of PPARα [24]. Increased de novo FAS inhibits carnitine palmitoyltransferase 1 (CPT1) transporter into mitochondria. Downregulation of CPT1 and PPARα reduces β-oxidation, causing lipid droplet accumulation and lipotoxicity [25]. Lipotoxicity damages mitochondrial membranes, further reducing ATP synthesis and triggering inflammasome activation in proximal tubules.

The shift from OXPHOS to aerobic glycolysis in PKD is an active driver of cystogenesis, not a bystander. This metabolic reprogramming supports proliferation but increases oxidative stress. Cystic cells are metabolically inflexible, offering a therapeutic vulnerability. Targeting this pathway with AMPK activators or glycolytic inhibitors has shown preclinical promise and deserves further clinical exploration.

### 2.2. Role of Mitochondrial Oxidative Stress in Renal Fibrosis and Cyst Formation

Mitochondrial oxidative stress plays a critical role in driving renal fibrosis and cyst formation in PKD through interconnected mechanisms. These include the direct activation of proliferative signaling, the release of mitochondrial DNA (mtDNA) that triggers cGAS-STING-dependent inflammation, and the promotion of fibrotic responses via TGF-β/Smad signaling [26,27]. Mitochondrial oxidative stress refers to the imbalance between the generation of reactive oxygen species (ROS) within mitochondria and the antioxidant defense system, leading to excessive accumulation of ROS and causing damage to mitochondria and cells [12].

#### 2.2.1. Sources of ROS Overproduction

PKD cells exhibit a shift toward aerobic glycolysis (Warburg effect), impairing mitochondrial oxidative phosphorylation and increasing ROS production [1,11].

Calcium signaling in PKD is a nexus of dysfunction, involving polycystins, endoplasmic reticulum–mitochondria crosstalk, and downstream oxidative stress and apoptosis. Endoplasmic reticulum (ER) is the largest pool of Ca^2+^ in cells. Regulated transfer of Ca^2+^ from the ER to mitochondria relate to inositol trisphosphate receptors (IP3Rs) and ryanodine receptors (RyRs) on the ER membrane and mitochondrial calcium uniporter (MCU) complex on the inner mitochondrial membrane. In PKD, inhibition of the phosphorylation of IP3R1 disrupts ER–mitochondria contact, impairing Ca^2+^ transfer, which reduces mitochondrial ATP production and increasing oxidative stress [28,29]. In ADPKD cells, the dysfunction of PC1, PC2, and other Ca^2+^ channels in the cytomembrane leads to mitochondrial Ca^2+^ overload and reduced cytosolic Ca^2+^ level and mitochondrial Ca^2+^ overload causes production of ROS [7,18,29].

Beyond calcium-mediated mechanisms, multiple pathways converge to amplify oxidative stress in PKD. The metabolic switch to aerobic glycolysis (Warburg effect) impairs OXPHOS efficiency, promoting electron leakage from complexes I and III. ROS-producing nicotinamide adenine dinucleotide phosphate hydrogen NAD(P)H-oxidase complex-4 (NOX4) is also a major source of oxidative stress. Research found that PCK rats have increased NOX-4-induced oxidative stress and mitochondrial abnormalities predominantly in cyst-lining tubular cells and renal endothelial cells [30]. In early PKD, NOX4-induced endothelial dysfunction may be an important contributor to cyst inflammation [23]. Fragmented mitochondria produce excess ROS and are less efficient in energy production [21,31]. Abnormal mitochondrial dynamics including increased fission and reduced fusion are observed in PKD, possibly driven by dysregulated dynamin-related protein-1 (DRP1) activity. Research showed that at the molecular level, downregulation of fusion proteins (MFN1, OPA1) and upregulation of pro-fission protein DRP1 create a fragmented mitochondrial network prone to oxidative damage [31]. As for antioxidant depletion, proteomics data demonstrated that there was about ~16% decrease in the abundance of most antioxidant enzyme systems in kidneys from mice with *PKD1* mutation, including superoxide dismutase (SOD) I and II, catalase, glutathione antioxidant system, and the majority of peroxiredoxins [26]. Sirtuins (SIRTs) play important roles in antioxidant pathways. SIRT1 and SIRT3, NAD^+^-dependent deacetylases are downregulated in PKD. Loss of SIRT3 deacetylates SOD2, reducing antioxidant defense and increasing superoxide in mitochondria [32].

#### 2.2.2. Consequences of ROS Overproduction

Elevated ROS can partially activate autophagy, and autophagy disorders further aggravate ROS production. Excessive ROS can destroy a variety of functional enzymes, mitochondrial proteins and mtDNA, resulting in the change in ATP synthesis and inducing mitochondrial membrane permeability transition [7]. Therefore, a vicious cycle of excessive ROS generation and impaired antioxidant defenses was formed, aggravating mitochondrial damage [30].

Mitochondrial ROS activate mTORC1 via PI3K/Akt, driving cyst epithelial proliferation. In human ADPKD cells, mitochondrial ROS increased ERK1/2 phosphorylation and decreased AMPK phosphorylation and ERK1/2 belongs to the mitogen-activated protein kinase superfamily that controls cell proliferation [30]. Suppressed AMPK activity regulates cystic fibrosis transmembrane conductance regulator (CFTR) channels, which is an ATP-gated chloride channel principally responsible for the secretion of cyst fluid [16,33]. Concurrently, ROS induce apoptosis in adjacent cells through BAX/BAK oligomerization, creating a pro-cystic microenvironment [34]. In addition, mitochondrial Ca^2+^ overload enhances cAMP and protein kinase A (PKA) signaling, upregulating the extracellular signaling-regulated kinase (ERK) pathway in cells derived from polycystic kidneys and cAMP-PKA activation also promotes excessive OXPHOS activity [21]. Due to increased mitochondrial superoxide production in *PKD1*-defective cells, the reduction in PGC-1α expression has been proposed to promote cyst proliferation [35]. In addition, miR-17 inhibits PPARα and PGC1α, which decreases OXPHOS, FAO, and mitochondria biogenesis, and results in ROS production and mitochondrial swelling [15].

Fibrosis is a prominent and progressive feature of ADPKD [36]. ROS can activate NOD-like receptor thermal protein domain associated protein 3 (NLRP3) inflammasome and upregulate the expression of cytokines IL-18, IL-1β, TGF-β, and NF-κB, resulting in renal fibrosis. Transforming growth factor beta TGF-β is a major fibrogenic cytokine, highly expressed in cystic epithelia in human ADPKD kidneys and cells. TGF-β activates Smad3 phosphorylation which is a hallmark of fibrosis. This stimulates myofibroblast transdifferentiation and collagen deposition [36].

ROS-induced mtDNA deletions impair Complex I and III, causing electron leakage and further ROS production. Research found upregulation of STING in *Pkd1* mutant mouse kidneys, which is triggered by cytosolic DNA. Damaged mtDNA escapes into the cytosol, activating cGAS-STING, which drives fibrotic gene expression and triggers the expression of inflammatory cytokines [30,37]. In *Pkd1* mutant mouse kidneys, targeting stimulator of interferon genes (STING) with its specific inhibitor decreases the expression of fibrotic markers, including TGF-β, α-SMA, collegen1 and fibronectin [37]. STING binds mitochondrial voltage-dependent anion channel 2 (VDAC2) via its transmembrane domain, disrupting ER–mitochondria contacts. STING inhibition restores mitochondrial structure and function, reduces inflammation and fibrosis, and induces p53-dependent apoptosis, delaying cyst progression [38]. However, how STING regulates the expression of fibrotic markers needs further exploration.

Recent studies have identified ferroptosis, an iron-dependent regulated cell death characterized by lipid peroxidation, as a contributor to PKD progression. ADPKD kidneys show downregulated glutathione peroxidase 4 (GPX4) and solute carrier family 7 member 11 (SLC7A11) with upregulated transferrin receptor 1 (TFR1), alongside mitochondrial cristae loss. Ferroptosis inducer erastin promotes cyst growth while inhibitor Fer-1 delays progression, indicating ferroptosis as a key mechanism of oxidative injury in PKD [39].

Mitochondrial oxidative stress serves as a hub linking metabolism, inflammation, and fibrosis in PKD. mtROS activates proliferative signaling, triggers mtDNA release and cGAS-STING inflammation, forming a self-amplifying loop. Antioxidants and STING inhibitors show efficacy in models, but careful titration is needed to avoid blocking beneficial redox signaling.

### 2.3. Role of Mitochondrial Autophagy in PKD

Autophagy is a cellular recycling process involving self-degradation and reconstruction of damaged organelles and proteins. Current evidence suggests that autophagy is critical in kidney physiology and homeostasis, related to apoptosis and cell cycle regulations. Evidences suggests that the ADPKD may be present with dysfunctional autophagy and multiple autophagic molecular parameters and signaling pathways are involved, primarily including mTOR and AMPK [40].

mTORC1 is the major negative regulator of autophagy, whereas AMPK and sirtuin 1 are positive regulators [41]. mTORC1 is a regulator of autophagy that is activated by amino acids or growth factors, such as insulin and insulin-like growth factor-1, via upstream pathways. Activation of mTORC1 suppresses autophagy through inhibitory phosphorylation of unc-51 like autophagy activating kinase 1 (ULK1), which is involved in autophagosome formation, especially in response to low availability of amino acids [42]. Hyperactivation of mTORC1 in PKD inhibits autophagy, leading to accumulation of damaged organelles and protein aggregates. Rapamycin, a mTOR inhibitor, restores autophagic flux and slows cyst growth in animal model, further suggesting that autophagy impairment has a pathogenic role in PKD. HIF-1α has been shown to upregulate both apoptosis and autophagy. HIF-1α is highly expressed in the late stages of PKD and the increase in HIF-1α is associated with an increase in LC3-II and beclin-1 [43]. Current study showed HIF-1α suppressing OXPHOS and promoting aerobic glycolysis. This Warburg effect depletes ATP, increases lactate, and starves mitophagy of energy [14].

Recent studies reveal that autophagy in ADPKD is complex and context-dependent. While mTORC1 hyperactivity suppresses basal autophagy, cystic epithelial cells show partial autophagic activation that often fails to clear damaged organelles [44]. Impaired autophagy correlates with mitochondrial dysfunction, including defective mitophagy, mtROS accumulation, and apoptosis [45]. Conversely, excessive TGFβ signaling downregulates the histone acetyltransferase MYST1 via SMAD3, epigenetically derepressing autophagy-related protein 7(ATG7) and BECLIN1 to drive autophagy-dependent myofibroblast differentiation and fibrosis [46]. Thus, impaired autophagy contributes to cystogenesis by inducing apoptosis, while excessive autophagy leads to fibrosis via epithelial–mesenchymal transition [44]. Autophagy in ADPKD thus acts as a double-edged sword: moderate autophagy clears dysfunctional mitochondria, whereas its impairment or overactivation accelerates cyst growth and fibrosis [44,45]. Future studies should dissect stage- and cell-type-specific autophagic pathways for targeted therapy.

Mitophagy serves as important component of mitochondrial quality controlled by removing impaired or dysfunctional mitochondria from the cell to warrant redox homeostasis and sustain cell viability [34]. A number of studies have emphasized the dysregulation of mitophagy in PKD [47]. Mitophagy is mainly mediated by microtubule-associated protein 1 light chain 3 (LC3)-associated autophagy receptors. Ubiquitin (Ub)-dependent mitophagy involves the mitochondrial serine/threonine protein kinase PINK1 and E3 Ub-protein ligase parkin (PINK1-parkin) pathway [41]. As one of the most common ciliopathies, PKD is thought to be associated with decreased mitophagy. In PKD, autophagy markers LC3 and beclin-1 are upregulated in the mouse model, suggesting autophagic flux dysregulation [43].

Additionally, PC1 and PC2 might play crucial roles in mitophagy. The C-terminal tail (CTT) of PC1 translocates to mitochondria. In the ADPKD mouse model, the C-terminal 200 amino acids of the large PC1 protein interacts with the mitochondrial enzyme Nicotinamide Nucleotide Transhydrogenase (NNT) to suppress cystic disease. This interaction modulates tubular and cyst cell proliferation and NNT is proved to be associated with apoptosis and proliferation [5]. Study showed that PC1 malfunction decreases the expression of proteins involved in Ca^2+^ uptake into the mitochondria in mouse model and enhanced Ca^2+^ uptake by the mitochondria is a key driver of cyst growth [48]. In addition, other studies proved that the elevated cytosolic Ca^2+^ overloads mitochondria via the mitochondria-associated endoplasmic reticulum membranes (MAMs), leading to autophagy and oxidative stress. Polycystin-2 (PC2, TRPP2), a non-selective cation channel, localizes to the ER and primary cilia. PC2 can directly bind the InsP_3_R and alter the distribution of the InsP_3_R subtypes within ER–mitochondrial contact sites [49]. In addition, current research shows that PC2 interacts with ER–mitochondrial tethering protein mitofusin-2 (MFN2) to regulate ER-mediated mitochondrial Ca^2+^ transfer. PC2 deficiency impairs mitochondrial Ca^2+^ buffering, leading to bioenergetic failure and apoptosis [50]. In PKD, loss of PC2 promotes increased ER-mitochondrial Ca^2+^ transfer and sensitivity to apoptosis, thus leading to apoptosis through mitochondrial depolarization, cytochrome c release and caspase-3 activation [48,50]. MAMs serve as a critical platform for mitophagy and impaired MAM integrity disrupts the initiation of mitophagy and exacerbates mitochondrial damage [51]. Cilia structure and function are also impaired due to polycystin deficiency, leading to dysregulated mitophagy [52]. In addition, in ARPKD, mutations in *PKHD1* alkalinize lysosomes block autophagosome–lysosome fusion and mitophagy [53]. Fibrocystin (FPC), the protein encoded by *PKHD1*, contains a mitochondrial localization signal in its C-terminal fragment ICD15 and translocates to mitochondria. Pkhd1 loss causes mitochondrial structural defects, while ICD15 reintroduction partially restores function. FPC and PC1 may synergistically suppress cilia-dependent cyst activation to maintain mitochondrial homeostasis [54].

**Figure 1:**
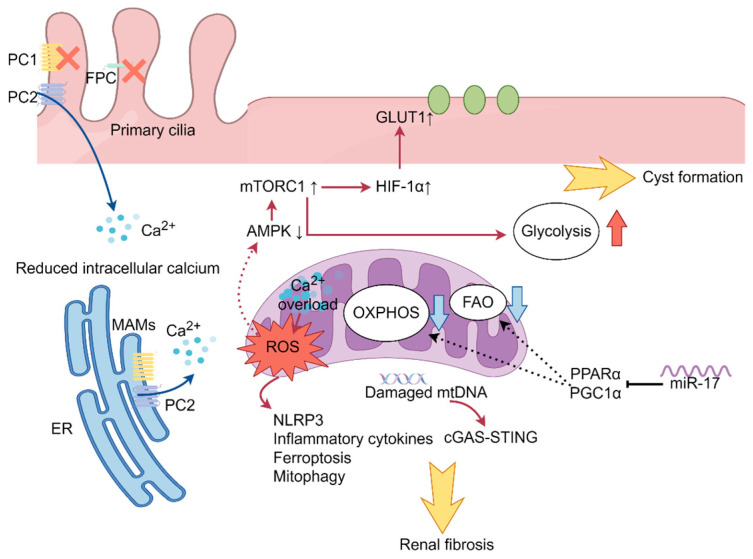
Mitochondrial dysfunction as a central hub in polycystic kidney disease (PKD) pathogenesis. This schematic illustrates the complex network of mitochondrial abnormalities that drive cyst formation and renal fibrosis in PKD. Loss of polycystin-1 (PC1), polycystin-2 (PC2), or fibrocystin (FPC) disrupts primary cilia signaling, leading to reduced intracellular calcium (Ca^2+^). AMP-activated protein kinase (AMPK) inactivation results in activation of the mechanistic target of rapamycin complex 1 (mTORC1) and hypoxia-inducible factor 1-alpha (HIF-1α), which upregulate glucose transporter 1 (GLUT1) and promote glycolysis. Simultaneously, oxidative phosphorylation (OXPHOS), fatty acid oxidation (FAO), and their key regulators peroxisome proliferator-activated receptor alpha (PPARα) and PGC1α are suppressed. MicroRNA-17 (miR-17) further impairs mitochondrial metabolism. Damaged mitochondria produce excessive reactive oxygen species (ROS) and release mitochondrial DNA (mtDNA), activating the NLRP3 inflammasome and the cGAS-STING pathway, which drives inflammatory cytokine production. These events promote ferroptosis, impair mitophagy, and ultimately lead to renal fibrosis and cyst formation. Disrupted mitochondria-associated endoplasmic reticulum membranes (MAMs) contribute to Ca^2+^ dysregulation and mitochondrial dysfunction.

Autophagy in PKD shows a paradox. mTORC1 hyperactivity inhibits basal autophagy. Yet cystic epithelial cells partly activate autophagic markers. Mitophagy is specifically impaired, causing damaged mitochondria to accumulate and ROS to increase. Restoring mitophagy, rather than general autophagy, may be a more precise approach. Interventions tailored to disease stage and cell type are needed for therapeutic benefit. To sum up, the complex network of mitochondrial dysfunction driving cystogenesis and disease progression in PKD is illustrated in Figure 1.

## 3. Mitochondrial-Targeted Therapeutic Strategies

Based on the probable role of mitochondria in PKD, the following mitochondrial-targeted therapeutic strategies have been supported by experimental evidence (Table 1).

### 3.1. Mitochondrial-Targeted Antioxidants

To reduce oxidative stress, studies demonstrated that overexpressing mitochondrial-targeted catalase or SS31 mitochondrial protective tetrapeptide improved metabolic derangements and reduced oxidative stress in *PKD1* mutant mice [26]. SS31, also known as MTP-131 or elamipretide, is a tetrapeptide that binds to cardiolipin in the inner mitochondrial membrane, enhances the efficiency of electron transfer and ATP production, and indirectly decreases ROS [7]. Moreover, SS31 was found to ameliorate the progression of ADPKD in a pregnant mouse model, without any observed teratogenic or harmful effect [55]. Alpha-lipoic acid (ALA), a sulphur-containing multifunctional antioxidant, was highlighted for its potential to mitigate oxidative damage in kidney diseases. The main disadvantage of ALA for ADPKD is that clinical evidence is limited to a single small, short-term study with no long-term renal endpoint data [56], and it has low oral bioavailability, a short half-life, potential toxicity at high doses, and rare case reports of membranous nephropathy [67]. Interestingly, melatonin was found to substantially reduce cysts in the *Drosophila* PKD model [68]. Recent studies demonstrate that melanin-like nanoparticles (MNPs) significantly ameliorate disease progression in a mouse model of ADPKD. Mechanistically, MNPs exert a dual action: they scavenge mitochondrial ROS and directly bind to the bZIP domain of cyclic AMP-responsive element-binding protein (CREB), preventing its interaction with cAMP-responsive elements (CRE) on genomic DNA and thereby inhibiting CREB-driven transcriptional activity. By targeting both ROS accumulation and aberrant CREB activation, two key branches of cystic epithelial dysfunction, MNPs represent a promising novel therapeutic strategy with considerable translational potential for ADPKD [57]. However, chronic safety remains unknown and MNPs treatment fails to correct several PKD-related transcriptional changes while unexpectedly altering other genes, raising concerns about potential off-target toxicity in a relatively benign disease.

### 3.2. Regulator of Mitophagy and Apoptosis

To regulate mitophagy, targeting at PC1 and PC2 expression is one of the therapies, and signaling pathways of AMPK and mTOR also contribute to mitophagy. The inhibitors of mTORC1 including rapamycin and rapamycin analogues could repair kidney function by regulating mitophagy. Metformin could affect cells and increase autophagy via the activation of AMPK and mitochondrial bioenergetics improvement [47]. In addition, sirtuins (SIRTs) regulate autophagic process by directly or indirectly deacetylating essential proteins of the autophagic machinery. Sirtuin-activating compounds (STACs) is a therapy, and resveratrol could appropriately activate SIRT1 to regulate mitophagy [32]. Long-term calorie restriction could also restore the autophagic activity via the activation of SIRT1 [47].

Acyl-CoA thioesterase 13 (ACOT13), a mitochondria-associated acyl-CoA thioesterase (Acot) gene, is reduced in ADPKD patients. Current evidence showed that high expression of ACOT13 triggered mitochondrial-dependent apoptosis in ADPKD cells, reducing cyst growth in vitro. ACOT13 may exert a protective role in ADPKD and may be a potential option for the treatment of ADPKD [69]. Other studies showed that second mitochondria-derived activator of caspases (Smac) mimetics can induce the death of TNF-α-dependent cystic renal epithelial cell, significantly reducing renal cysts in mouse models [70]. In a PKD *Drosophila* model, the Smac mimics affected different tubular regions differentially and different Smac mimics may make personalized pharmacological treatments possible [66]. Current research showed targeting STING with its specific inhibitor such as C-176, delays cyst growth in several PKD mouse models restrained mitochondrial structure and function by inducing a p53-dependent apoptosis, demonstrating STING is also a novel therapeutic target for ADPKD treatment [37].

### 3.3. Calcium Signaling Modulation

Based on the calcium signaling modulation, Ca^2+^ uptake inhibitors could potentially be used for PKD. Mitochondrial Ca^2+^ uniporter (MCU) and voltage-dependent anion channels 1 and 3 (VDAC) were downregulated in ADPKD mouse models and cells. A relevant research showed that MCU inhibitor Ru360 could inhibit cyst growth and alter both apoptosis and cell proliferation and the cystic fibrosis (CFTR) corrector VX-809 could reverse Ca^2+^ signaling, identifying novel therapeutic targets for treating ADPKD [48]. Selective calcium-sensing receptor (CaSR) activation restores mitochondrial calcium content, rescuing mitochondrial energy status in human conditionally immortalized proximal tubular epithelial cells (ciPTEC) stably knocked down for PC1, which making CaSR a possible candidate as a therapeutic target [28].

TRPP2 and TRPC3 channels are also involved in the mitochondrial calcium regulation. For patients with *PKD2* mutations, PC2 is a potential therapeutic target [50]. Transient receptor potential channel, subtype C, member 3 (TRPC3) is a non-selective cation channel modulating calcium signals. TRPC3 upregulation upon PC2 knockdown aggravated the mitochondrial abnormalities and cell proliferation by modulating mitochondrial calcium uptake [71]. By correcting dysregulated mitochondrial calcium influx, TRPP2 and TRPC3 channel inhibitor may be a promising therapy for ADPKD treatment.

### 3.4. Metabolic Reprogramming Interventions

Previous studies highlighted the critical role for transcription factor (TF) GLI-Similar 3 (GLIS3) in the development of PKD. A current study demonstrated that GLIS3, in coordination with hepatocyte nuclear factor 1 beta (HNF1B) and nuclear respiratory factor 1 (NRF1), regulates mitochondrial metabolism [72]. Its activation reversed metabolic defects in PKD, having a protective effect against cyst formation. GLIS3 modulators may have potential for future therapeutic strategies.

In PKD, hyperactivation of mTORC1 and downregulation of AMPK create a metabolic imbalance that drives cyst growth. Targeting this axis, mTOR inhibitors and AMPK activators have been explored. Rapamycin inhibits mTORC1 by binding FKBP12. Preclinical studies show reduced cyst growth, but clinical trials report limited efficacy due to compensatory mTORC2 activation and off-target effects [16].

Among AMPK activators, metformin, salsalate, 2-deoxy-D-glucose (2DG), and ketogenic dietary interventions each act through distinct mechanisms [14]. Metformin indirectly activates AMPK by inhibiting mitochondrial complex I and inhibits the ERK pathway, thereby suppressing CFTR and mTORC1 to reduce cyst growth [73]. Metformin offers practical advantages, including oral availability, low cost, and a well-established safety profile from decades of use. Its main limitations are gastrointestinal intolerance, and additional clinical trial data are needed to fully validate its long-term efficacy and safety in ADPKD patients [59,73]. In contrast, salsalate is a direct AMPK activator that binds the AMPK β1 isoform and prevents dephosphorylation of Thr172 [60]. This leads to phosphorylation of acetyl-coenzyme A (CoA) carboxylase (ACC) to enhance fatty acid oxidation, suppression of mTORC1 and NF-κB, and restoration of PGC1α expression, reducing proliferation and inflammation without affecting cAMP [61]. The glucose analog 2-deoxy-D-glucose directly targets the upregulated Warburg effect in cystic epithelial cells by inhibiting glycolysis. Early 2-DG treatment reduced kidney cyst index and suppressed proliferation in *Pkd1* mutant mice [62]. Dietary interventions that reduce carbohydrate intake induce ketosis, producing β-hydroxybutyrate which activates AMPK, suppresses mTORC1, and enhances fatty acid oxidation [74]. However, long-term adherence is poor, and risks of micronutrient deficiencies, elevated blood lipids and nephrolithiasis remain unaddressed [75]. Therefore, larger and longer trials are needed before routine clinical recommendation.

As a modulator of AMPK-driven pathways, semaglutide delays ADPKD cyst progression by upregulating mitochondrial respiratory chain genes (mt-Nd6, mt-Cytb), improving mitochondrial structure, inducing ketosis (BHB) and AMPK activation, enhancing fatty acid oxidation (CPT1A, ACOX1), and suppressing TGF-β/α-SMA fibrosis, highlighting its mitochondria-targeted therapeutic potential [76].

miR-21 is upregulated in PKD kidneys, inhibiting PPARα and activating SIRT1, leading to mitochondrial dysfunction and increased superoxide production. Anti-miR-21 treatment restores PPARα, suppresses SIRT1, improves mitochondrial function, and reduces cyst growth, highlighting miR-21 targeting as a promising metabolic intervention [65].

What is more, mitochondrial transcription factor A (TFAM) is a nuclear-encoded factor, regulating replication and transcription of the mitochondrial genome. Research showed that TFAM deficiency and mitochondria depletion are common in PKD tissues and TFAM deficiency exacerbated cystic disease [35]. The transcription factor GLIS3 directly activates TFAM transcription, positioning TFAM as a downstream effector of the GLIS3-HNF1B-NRF1 network [72]. Restoring TFAM could mitigate mitochondrial depletion which makes TFAM a promising target for PKD.

## 4. Challenges and Future Directions

Specific pathophysiological mechanisms of mitochondrial dysfunction in PKD still require further exploration. For example, dysregulated mitochondrial calcium signaling driven by defective PC1 and PC2 contributes to apoptosis and cystogenesis [18,50]. However, restoring calcium balance is challenging because calcium plays dual roles in both cell survival and death, necessitating precise modulation [49]. Moreover, the precise molecular functions of PC1 and PC2 at mitochondria-associated membranes remain incompletely defined. Beyond ADPKD, the rarer but more severe ARPKD also demands in-depth investigation into the role of mitochondrial dysfunction in its pathogenesis.

As for the mitochondrial-targeted therapeutic strategies, developing more precise and selective drugs to minimize side effects is the main target and a major challenge. While antioxidants like elamipretide show efficacy in mice [26], translating these findings to humans requires overcoming challenges in drug delivery and minimizing off-target effects. Mitochondrial ROS overproduction accelerates tubular injury and fibrosis [21]. Developing compounds like MitoQ or SS-31 to scavenge ROS specifically in mitochondria, exploring small molecules to stabilize mitochondrial dynamics and improve bioenergetics are future directions [31].

While challenges remain in targeting mitochondrial pathways with precision, emerging strategies ranging from organelle-specific antioxidants to metabolic modulators hold transformative potential. Future research must bridge gaps between preclinical models and human biology, emphasizing personalized approaches to mitigate this complex disease.

## 5. Conclusions

Advances in understanding mitochondrial biology in PKD underscore its centrality in both ADPKD and ARPKD pathogenesis, bridging genetic mutations to cellular dysfunction. Mitochondrial dysfunction in PKD manifests through altered energy metabolism, oxidative stress, impaired mitophagy, and disrupted cellular signaling, all of which drive cystogenesis and disease progression. Given this central role, targeting mitochondrial pathways via metabolic modulators, antioxidants, or calcium regulators offers promising therapeutic avenues to slow or halt PKD.

## Figures and Tables

**Table 1:** Summary of key mitochondrial targets and agents.

Strategy	Key Targets/Agents	Research Stage	References
Antioxidants	SS31(Elamipretide)	Preclinical	[26,55]
	ALA	Unregistered short-term clinical study (Phase II)	[56]
	melanin-nanoparticles	Preclinical	[57]
Metabolic Reprogramming	Metformin	NCT02656017 (Phase II) NCT02903511 (Phase II)	[58,59]
	Salsalate	Preclinical	[60,61]
	2-Deoxy-D-glucose (2DG)	Preclinical	[62]
	dietary interventions	NCT04680780 (exploratory, phase II-like feasibility study) NCT04534985 (Phase II)	[63,64]
	Anti-miR-21-SNA	Preclinical	[65]
Mitophagy Enhancement	Resveratrol (SIRT1 activator)	Preclinical	[32]
Apoptosis Induction	Smac Mimetics	Preclinical	[66]
	STING inhibitors	Preclinical	[37]
Calcium Regulation	MCU inhibitor (Ru360)	Preclinical	[48]
	CaSR agonists	Preclinical	[28]
Gene Therapy	TFAM	Preclinical	[35]

## Data Availability

No new data were created or analyzed in this study. Data sharing is not applicable to this article.

## Abbreviations

The following abbreviations are used in this manuscript:

ACOX1	Acyl-CoA oxidase 1
ADPKD	Autosomal dominant polycystic kidney disease
AMPK	AMP-activated protein kinase
ARPKD	Autosomal recessive polycystic kidney disease
BHB	Beta-hydroxybutyrate
CaSR	Calcium-sensing receptor
cGAS	Cyclic GMP-AMP synthase
CPT1A	Carnitine palmitoyltransferase 1A
CTT	C-terminal tail
DKD	Diabetic kidney disease
DRP1	Dynamin-related protein 1
ER	Endoplasmic reticulum
FAO	Fatty acid oxidation
FPC	Fibrocystin/polyductin
GLUT1	Glucose transporter 1
GPX4	Glutathione peroxidase 4
HIF-1α	Hypoxia-inducible factor 1-alpha
ICD15	Intracellular domain 15 (FPC C-terminal fragment)
IP3R	Inositol 1,4,5-trisphosphate receptor
MAM	Mitochondria-associated endoplasmic reticulum membrane
MCU	Mitochondrial calcium uniporter
MFN2	Mitofusin 2
mTORC1	Mechanistic target of rapamycin complex 1
NF-κB	Nuclear factor kappa-B
NLRP3	NOD-like receptor family pyrin domain containing 3
NNT	Nicotinamide nucleotide transhydrogenase
OXPHOS	Oxidative phosphorylation
PC1	Polycystin-1
PC2	Polycystin-2
PGC1α	Peroxisome proliferator-activated receptor gamma coactivator 1-alpha
PKD	Polycystic kidney disease
PPARα	Peroxisome proliferator-activated receptor alpha
ROS	Reactive oxygen species
SIRT1	Silent information regulator 1
SLC7A11	Solute carrier family 7 member 11 (xCT)
STING	Stimulator of interferon genes
TFAM	Mitochondrial transcription factor A
TFR1	Transferrin receptor 1
TGF-β	Transforming growth factor-beta
VDAC	Voltage-dependent anion channel
